# In-hospital Cardiac Arrest Following Spinal Cord Injury: A Scoping Review

**DOI:** 10.1298/ptr.E10329

**Published:** 2025-03-15

**Authors:** Daisuke KUBO, Tatsuya OKAWA

**Affiliations:** 1 Department of Health Data Science, Yokohama City University, Japan; 2 Department of Rehabilitation Services, Tokai University Hospital, Japan

**Keywords:** Heart arrest, Cardiopulmonary resuscitation, Quadriplegia

## Abstract

Objectives: This review aimed to examine the characteristics of patients with spinal cord injury (SCI) who experience in-hospital cardiac arrest (IHCA), as well as the timing, circumstances, and interventions associated with these events. Methods: A comprehensive literature search was conducted across multiple databases, including PubMed, Scopus, Cochrane Library, and Igaku Chuo Zasshi Web (in Japanese), for studies published up to 2024. Two independent reviewers screened the literature. Data were extracted from the selected studies regarding the characteristics of patients with SCI who experienced IHCA, the timing of IHCA, the circumstances under which it occurred, and the interventions provided. Results: A total of 56 studies met the inclusion criteria. IHCA most commonly occurs in patients with complete cervical SCI. The time frame for IHCA occurrence ranged from 1 day and 2.5 months post-injury. IHCA frequently occurs during endotracheal suctioning or postural changes. The most commonly reported intervention for IHCA was the use of a pacemaker. Conclusions: In patients with SCI, IHCA is more prevalent among those with severe cervical injuries and is often triggered by procedures such as suctioning or postural adjustments. Physical therapist needs to implement robust risk management strategies. These findings are crucial for both clinical practice and future research.

## Introduction

In-hospital cardiac arrest (IHCA) is a serious cardiovascular complication that can occur following acute spinal cord injury (SCI)^1–4)^, and it is primarily caused by disturbances in the autonomic nervous system^5)^ and is associated with increased mortality in patients with SCI^6)^. Previous studies have shown that patients with SCI who experience IHCA have a significantly higher odds ratio (22.4) for in-hospital mortality compared to those without IHCA^6)^. Therefore, understanding IHCA in patients with SCI is essential for the clinical practice of professionals involved in rehabilitation.

Studies have reported that IHCA occurs in approximately 0.6% of all patients with SCI^3)^ and in 16.0% of those with severe cervical cord injuries^1)^. However, these studies provide limited information on the timing of IHCA onset, the circumstances surrounding it, and the interventions used. This gap in information hinders the ability to predict IHCA in clinical settings and to provide appropriate care.

To address this gap, a scoping review synthesizing existing studies is recommended. This approach allows for a comprehensive assessment of previous findings and offers suggestions regarding the timing, situations, and prevention of IHCA following acute SCI. Using the Population, Concept, Context (PCC) framework, this study aimed to conduct a scoping review of available literature to provide comprehensive and practical suggestions for the management of IHCA in patients with SCI.

## Methods

This scoping review adopted the methodology outlined by Arksey and O'Malley^7)^ and was conducted in accordance with the Preferred Reporting Items for Systematic Reviews and Meta-Analyses Extension for Scoping Reviews (PRISMA-ScR) guidelines. The review aimed to identify knowledge gaps and understand overall trends, which is why a formal quality assessment of the studies was not performed. No review protocols have been registered for this study.

### Identifying the research question

The PCC framework was used as recommended in scoping reviews. The population consisted of patients diagnosed with SCI. The concept focused on the characteristics of IHCA, including the severity of illness, the timing and circumstances of IHCA, and preventive strategies. The context was defined as the acute care setting, specifically within the first 6 months following hospitalization.

The review aimed to address the following research questions: (1) What is the patient profile for IHCA following SCI?; (2) When does IHCA occur after SCI?; (3) Under what circumstances does IHCA occur in patients with SCI?; and (4) What strategies can prevent IHCA in individuals with SCI?

### Information source and search strategy

A systematic search was conducted across the following databases: PubMed, Scopus, Cochrane Library, and Igaku Chuo Zasshi (ICHUSHI) Web (in Japanese) on December 6, 2024. The search used a combination of search terms related to SCI and IHCA (Appendix 1). Studies were included based on the following criteria: (1) investigations involving patients with SCI; (2) participants aged 18 years or older; and (3) studies that explicitly described IHCA. For studies that included various neurological conditions or broad age groups, only data specific to patients with SCI aged 18 years and above were considered.

Exclusion criteria included studies involving non-human participants, SCI in the chronic phase (>6 months from injury), individuals under 18 years of age, IHCA occurring during surgery, and articles that were not accessible. Review papers, letters to the editor, bulletins, and conference abstracts were excluded.

The literature review was conducted independently by 2 reviewers (DK and TO), and any disagreements were resolved through discussion. In the event of a disagreement between the 2 reviewers, a third reviewer (MK) was consulted to facilitate the resolution process and finalize the selection of the literature.

### Data extraction and synthesis

Two researchers initially compiled summaries of essential data from the selected studies, including the first author’s name, publication year, study location (country), study design, cause of injury, and patient characteristics such as injury severity and level. Subsequently, data regarding the incidence of IHCA, timing, setting, and circumstances leading to IHCA onset were extracted. For studies that included patients with both with and without IHCA, only data specific to those who experienced IHCA were considered. Studies that could not determine the time interval between the injury and the onset of IHCA were excluded from the analysis of the number of days. Any disagreements between the 2 researchers were resolved through discussion, with a third researcher (MK) consulted if necessary to resolve any remaining disputes.

## Results

**Fig. 1.**
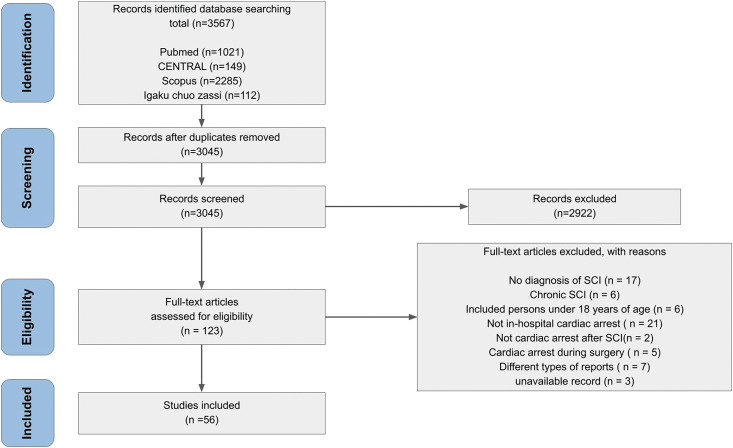
Flow diagram SCI, spinal cord injury

We identified 56 articles^2,4–6,8–59)^ that met the inclusion criteria for this review (Fig. 1). The appendix summarizes the study population and design of each article (Appendix 2). Of the 56 articles, 40 were case reports^9–17,19,21–27,29,32,34–36,38–45,47–51,53,56–59)^, 15 were observational studies^2,4–6,8,18,20,28,30,31,33,37,46,52,55)^, and 2 were intervention studies^40,54)^ (There was 1 document that included elements of both a case report and an interventional study). A total of 81 patients were reported to have experienced IHCA following SCI^5,8–30,32–59)^. Table 1 provides detailed information on these cases, including the timing, circumstances, and outcomes of IHCA. Injury severity was documented in 45 patients^9–12,14–18,20,22,25,26,28,29,32–37,40,43–45,47,48,50–53,58,59)^, with 33 classified as complete injuries and 12 as incomplete. Injury levels were recorded for 56 patients^9,10,13,14,16–19,21–29,32–51,53,55–59)^, of whom 43 had cervical, 11 had thoracic, and 3 had lumbar SCI. The time from injury to IHCA ranged from 1 day to 2.5 months^9–13,15–17,19,21–23,26,27,29,32,33,35,36,38–45,47,48,50,51,53,56–59)^. The location of IHCA was reported in 14 articles^9,10,22,23,26,27,33,35,42,48,51–53,56)^, with 9 incidents occurring in intensive care units, 4 in non-intensive care settings, and 1 in both settings^56)^. In 32 cases, descriptions of the situations surrounding IHCA were not provided. Among the remaining cases, IHCA was most commonly associated with suctioning^13,17,25,36,37,40,56)^ and postural changes^9,10,13,25,42)^. Other situations included anesthesia administration^35,38,39,54)^, tracheostomy^9,40)^, urinary catheterization^29)^, manual muscle testing^43)^, transportation^44,57)^, bronchoscopy^48)^, extubation^18)^, manual abdominal compression^17)^, recruitment maneuver,^56)^ and defecation^56)^. Interventions for IHCA included pharmacological treatments, surgical interventions, and other measures. Medications administered included atropine^17,29,36,37,40,44–46,56)^, epinephrine^9,25,36)^, ephedrine^50)^, dopamine^9,11)^, catecholamines^59)^, droxidopa^56)^, heparin^15,57,59)^, rivaroxaban^57)^, theophylline^11)^, alteplase^15)^, probanthine^36)^, midodrine^9)^, isoproterenol^45)^, cilostazol^9)^, aminophylline^51)^, lidocaine^58)^, and magnesium sulfate^58)^. Surgical interventions included pacemaker^10,11,13,16,17,19,28,33,42,44–47,50–52,56)^, embolectomy^15)^, thrombectomy^57,59)^, and inferior vena cava filter placement^15,19)^. Additional measures included mechanical ventilation^57)^, percutaneous cardiopulmonary support^59)^, antithrombin III replacement therapy^59)^, anti-hyperthermia^59)^, oxygen inhalation^36,37)^, and avoidance of succinylcholine^38)^.

**Table 1.** Profile of patients with SCI who developed IHCA

First author	Patients with IHCA n (%)	The severity of patients with IHCA	Injury level of patients with IHCA	Timing	Situation	Treatments	Patients outcome
Okuda A	7 (16.7)	NR	NR	NR	NR	NR	NR
Hachiya S	1 (100)	Complete = 1	C3 = 1	Day 26	Postural change	Pharmacotherapy Epinephrine Dopamine Midodrine Cilostazol	Transfer = 1
Murakami T	1 (100)	Incomplete = 1	C3–5 = 1	Days 29 and 32	Postural change	Pacemaker	Transfer = 1
Miura N	1 (100)	Complete = 1	NR	Days 11 and 22	NR	Pharmacotherapy Dopamine Theophylline Pacemaker	Transfer = 1
Inoue S	1 (100)	Incomplete = 1	NR	Day 1	Tracheostomy	NR	Home = 1
Onuki T	1 (100)	NR	C3–4 = 1	Day 16	Postural change Suctioning	Pacemaker	NR
Saruyama M	1 (100)	Complete = 1	C6 = 1	Postoperative 6 weeks (42 days)	NR	NR	Death = 1
Kiuchi S	1 (100)	Incomplete = 1	NR	Day 44	Transfer (To bed from wheelchair)	Pharmacotherapy Unfractionated Heparin Alteplase Embolectomy Inferior vena cava filter	NR
Yamada Y	1 (100)	Complete = 1	C5 = 1	Days 5, 13, and 21	NR	Temporary pacing Pacemaker	NR
Lee J	2 (100)	Complete = 1 Incomplete = 1	C6 = 2	Case 1 = Day 27 Case 2 = Day 30	Suctioning Abdominal compression	Pharmacotherapy Atropine sulfate Pacemaker	Transfer = 2
Nagayama M	1 (2.3)	Complete = 1	C6 = 1	NR	Extubation	NR	Death = 1
Meredith A	2 (50)	NR	C3–4 = 1 C5–6 = 1	Case 1 = Day 2 Case 2 = Day 23	NR	Pacemaker	Home = 1 Death = 1
Ull C	6 (10.0)	Complete = 6	NR	NR	NR	NR	NR
Lee SY	1 (100)	NR	C3–4 = 1	Day 9	NR	NR	Death = 1
Benton JA	1 (100)	Complete = 1	C4 = 1	Day 25	NR	NR	Death = 1
Marion TE	NR	NR	NR	NR	NR	NR	NR
Ijmkers S	1 (100)	NR	C1–2 = 1	Day 3	NR	Pharmacotherapy Inotropic drug	Death = 1
Riley K	1 (100)	NR	C5–6 = 1	NR	NR	NR	Death = 1
Oh Y-M	1 (100)	Complete = 1	T4 = 1	Postoperative 2 day	Postural change Suctioning	Pharmacotherapy Epinephrine (Before postural change and suctioning)	Home = 1
Wu Y-S	1 (100)	Complete = 1	C4–5 = 1	Day 1	NR	NR	Death = 1
Efeoglu M	1 (100)	NR	C2–7 = 1	Day 1	NR	NR	Death = 1
Bartholdy K	3 (10.0)	Complete = 3	C1–2 = 3	NR	NR	Pacemaker	NR
Jain A	1 (100)	Complete = 1	T9 = 1	2 weeks (14 days)	Urinary catheterization	Pharmacotherapy Atropine	Death = 1
Dididze M	2 (5.7)	NR	NR	NR	NR	NR	NR
Babu R	NR	NR	NR	NR	NR	NR	NR
Angthong C	1 (100)	Incomplete = 1	C6 = 1	Day 15	NR	NR	Death = 1
Ruiz-Arango AF	2 (2.7)	Complete = 2	C4–5 = 1 C3–5 = 1	Case 1 = Days 5, 26, 27, and 63 Case 2 = Day 8	NR	Pacemaker	Death = 1 NR = 1
Chen D	NR (0.7)	NR	NR	NR	NR	NR	NR
van den Bout AH	1 (100)	Complete = 1	C4 = 1	NR	NR	NR	Death = 1
Brooke MM	3 (100)	Incomplete = 1 NR = 2	T11 = 2 L2 = 1	Case 1 = 6 weeks (42 days) Case 2 = 2.5 months Case 3 = 7 weeks (49 days) and 9 weeks (63 days)	Succinylcholine injection Intubation	NR	NR
Mathias CJ	2 (50)	Complete = 2	C3–4 = 1 C4 = 1	Case 1 = Days 39, 40, 41, and 44 Case 2 = Day 2	Suctioning	Pharmacotherapy Probanthine Epinephrine Atropine Oxygen inhalation	NR
Welply NC	2 (28.6)	Complete = 2	C3–4 = 1 C4 = 1	NR	Suctioning	Pharmacotherapy Atropine Oxygen inhalation	NR
Snow JC	1(100)	NR	L4 = 1	Day 27	Succinylcholine injection Intubation	Avoidance of succinylcholine	Home = 1
Baker BB	1(100)	NR	L1–4 = 1	Day 45	Succinylcholine injection	NR	NR
Dollfus P	1(12.5)	Incomplete = 1	C4 = 1	Day 16	Tracheostomy Suctioning	Pharmacotherapy Atropine	Death = 1
Wu Y	1 (100)	NR	C1–4 = 1	Day 1	NR	NR	NR
Kim SW	1 (100)	NR	C4–7 = 1	Day 22	Postural change	Pacemaker	NR
Malmqvist L	4 (8.0)	NR	NR	NR	NR	NR	NR
Kovindha A	1 (100)	Incomplete = 1	T11 = 1	Day 18	Manual muscle test	NR	Death = 1
Kumagai N	1 (100)	Incomplete = 1	C4–7 = 1	Day 1	During transportation	Pharmacotherapy Atropine Pacemaker	Transfer = 1
Veeravagu A	NR	NR	NR	NR	NR	NR	NR
Peyrol M	1 (100)	Complete = 1	T2 = 1	Day 6	NR	Pharmacotherapy Atropine Isoproterenol Pacemaker	NR
Moerman JR	2 (1.8)	NR	C2–6 = 1 C5–7 = 1	NR	NR	Pharmacotherapy Atropine Pacemaker	Transfer = 1 Death = 1
Singh A	1 (100)	Incomplete = 1	C5–6 = 1	Day 10	NR	Pacemaker	NR
Sobiech S	1 (100)	Complete = 1	C3–4 = 1	Day 23	Bronchoscopy	NR	Death = 1
Velnar T	1 (100)	NR	C3 = 1	NR	NR	NR	Transfer = 1
Sanghvi AV	1 (100)	Complete = 1	C7–T1 = 1	Days 8, 10, 35, and 62	NR	Pharmacotherapy Ephedrine Pacemaker	NR
Weant KA	1 (100)	Complete = 1	T4 = 1	Days 21 and 22	NR	Pharmacotherapy Aminophylline Pacemaker	Transfer = 1
Franga DL	2 (16.7)	Complete = 2	NR	NR	NR	Pacemaker	Death = 2
Bhuiyan MS	1 (100)	Incomplete = 1	T4 = 1	Day 17	NR	NR	Death = 1
Tobey RE	1 (25.0)	NR	NR	NR	Succinylcholine injection	NR	NR
Gardner BP	4 (10.0)	NR	C4 = 1 C5 = 2 C6 = 1	NR	NR	NR	Death = 2 Home = 2
Yamanaka T	1 (100)	NR	C4–7 = 1	Days 7, 15, 16, and 17	Suctioning Recruitment maneuver During defecation	Pharmacotherapy Atropine Droxidopa Pacemaker	Transfer = 1
Chikaishi N	1 (33.3)	NR	T5/6 =1	Day 18	During transportation	Mechanical ventilation Thrombectomy Pharmacotherapy Heparin Rivaroxaban	NR
Funayama T	1 (100)	Incomplete = 1	T1 = 1	Day 4	NR	Pharmacotherapy Catecholamines Heparinization Percutaneous Cardiopulmonary support Antithrombin III replacement therapy Thrombectomy Inferior vena cava filter Anti-hyperthermia	Transfer = 1
Mahanta DS	1	Complete = 1	C5–6 = 1	Day 1	NR	Pharmacotherapy Lidocaine Magnesium sulfate	Home = 1

SCI, spinal cord injury; IHCA, in-hospital cardiac arrest; NR, not reported

## Discussion

This comprehensive scoping review methodically examined and assessed existing research on IHCA following SCI. Key findings include the following: (1) IHCA was predominantly observed in patients with complete and cervical SCI; (2) IHCA occurred between 1 day and 2.5 months post-injury; (3) IHCA most frequently occurred during endotracheal suctioning, followed by postural changes; and (4) Pacemaker implantation was the most commonly reported intervention for IHCA.

### Profile for IHCA following SCI

Previous studies have highlighted the correlation between the level of SCI and the incidence of IHCA. In a study involving 71 patients with acute SCI, IHCA was observed only in those with severe cervical injuries, with no cases reported in patients with thoracic SCI^1)^. Furthermore, IHCA was limited to patients with complete motor function loss associated with cervical cord injury^1)^. From these findings, it can be inferred that the severity of injury, particularly complete SCIs, is a more significant factor in the development of IHCA than incomplete injuries. Our pooled results support previous reports that highlight the importance of both cervical cord injury and injury severity as key factors related to IHCA following SCI.

The underlying mechanism through which individuals with complete cervical SCI develop IHCA is posited to originate from dysregulation in the autonomic nervous system. Research analyzing heart rate variability in 13 patients with SCI revealed a diminution in low-frequency power and amplitude among those with cervical SCIs, compared to healthy individuals. Conversely, high-frequency power and amplitude were comparable to those observed in healthy individuals^60)^. Given these findings, incorporating this demographic into future research focusing on IHCA after SCIs is deemed imperative.

### Timing of IHCA following SCI

Previous studies have not provided adequate information regarding the timing of IHCA in the acute phase post-SCI^1)^. Although studies involving patients with acute-phase SCI frequently document early cardiovascular abnormalities during hospitalization, they lack specific details regarding the timing of IHCA onset^2,4–6,8,18,20,24,28,30,31,34,37,46,49,52,54,55)^. This review synthesizes data from individual case reports, revealing that IHCA can occur as early as the day of injury and up to 2.5 months post-injury. These findings offer valuable insights for the design of future observational studies concerning IHCA post-SCI. In addition, rehabilitation professionals may need to take into account the potential occurrence of IHCA when planning interventions during the first 2.5 months post-injury.

### Occurrence of IHCA

This review highlights that IHCA often occurs during situations such as suctioning and postural change, which are performed as part of respiratory rehabilitation. Therefore, professionals involved in rehabilitation need to be aware of the potential occurrence of IHCA when performing suctioning and postural changes. However, a specific description of the postural change could not be identified. This review also revealed that information regarding the situations of IHCA occurrences was not reported in 31 articles^2,4–6,8,11,14,16,19–22,24,26–28,30–34,41,45–47,49–53,55)^, indicating a lack of information. This suggests a deficiency in crucial information needed for risk management in the acute rehabilitation of SCI patients. Future research should focus on identifying the circumstances under which IHCA occurs.

### Intervention to the IHCA

No intervention studies were identified for IHCA following SCI. This may be due to the rarity of IHCA in patients with SCI, with previous reports indicating an occurrence rate of 0.6%^3)^. The infrequency of IHCA poses challenges in conducting prospective studies, as rare outcomes require large sample sizes. Additionally, 27 articles in this review did not report any interventions^2,4–6,8,12,14,18,20–22,24,26,27,30–32,34,35,39,41,43,48,49,53–55)^. Among the cases that did, pacemaker implantation was the most commonly cited intervention. Given its effectiveness, pacemaker implantation was frequently mentioned in the studies reviewed. The included case reports also detailed cases where sufficient oxygen therapy effectively prevented bradycardia and cardiac arrest^36,37)^. Therefore, rehabilitation professionals should facilitate interprofessional communication to ensure the appropriate implementation of pharmacological therapies, pacemaker interventions, and oxygen therapy.

## Conclusions

Currently, there is a lack of sufficient observational studies with adequate sample sizes to provide a comprehensive understanding of IHCA in patients with acute SCI requiring acute care. Therefore, further observational studies are needed to address this knowledge gap. Additionally, it is important to acknowledge that patients with SCI within the first 2.5 months post-injury are at heightened risk for IHCA. Future studies should consider this timeframe when designing observational studies on this topic.

## Acknowledgements

We wish to acknowledge Masato Kaneko from the Department of Health Data Science at Yokohama City University for serving as the third reviewer and assisting with the literature screening. We are also grateful to the staff at Tokai University Hospital for their valuable advice, and we extend our deepest thanks to all collaborators for their support.

## Funding

This research was funded by the Kanagawa Physical Therapy Association.

## Conflicts of Interest

The authors have no conflicts of interest to declare.

## Supplementary Materials

Appendix.1 Search strategy (Pubmed)

Appendix.2 Characteristics of included studies.
