# Supplementary material for: In-hospital Cardiac Arrest Following Spinal Cord Injury: A Scoping Review
Source: Phys Ther Res. 2025 Mar 15;28(1):68–75. doi: 10.1298/ptr.E10329 (PMC12047041; doi:10.1298/ptr.E10329)
Supplement: Appendix.1 — Search strategy (Pubmed) [file ptr-28-68-s01.pdf]

Appendix.1 Search strategy (Pubmed)

Number	Searching terms
#1	Heart arrest[MeSH Terms]
#2	asystolic [Title/Abstract]
#3	"In-hospital cardiac arrest"[Title/Abstract]
#4	arrest[Title/Abstract]
#5	Cardiopulmonary resuscitation[Title/Abstract]
#6	#1 OR #2 OR #3 OR #4 OR #5
#7	Spinal cord injuries[MeSH Terms]
#8	Spinal cord ischemia[MeSH Terms]
#9	central cord syndrome[MeSH Terms]
#10	"Paraplegia"[Mesh]
#11	"Quadriplegia"[Mesh]
#12	"Brown-Sequard Syndrome"[Mesh]
#13	"Spinal cord injur*"[Title/Abstract]
#14	"spinal cord ischem*"[Title/Abstract]
#15	"central cord syndrome"[Title/Abstract]
#16	"central cord injury syndrome"[Title/Abstract]
#17	parapleg*[Title/Abstract]
#18	Quadripleg*[Title/Abstract]
#19	tetrapleg*[Title/Abstract]
#20	"Brown sequard syndrome"[Title/Abstract]
#21	"Brown-sequard syndrome"[Title/Abstract]
#22	"Cervical Spine"[Title/Abstract]
#23	"thoracic Spine"[Title/Abstract]
#24	"lumbar Spine"[Title/Abstract]
#25	SCI[Title/Abstract]
#26	#7 OR #8 OR #9 OR #10 OR #11 OR #12 OR #13 OR #14 OR #15 OR #16 OR #17 OR #18 OR #19 OR #20 OR #21 OR #22 OR #23 OR #24 OR #25
#27	#6 AND #26
