# Supplementary material for: In-hospital Cardiac Arrest Following Spinal Cord Injury: A Scoping Review
Source: Phys Ther Res. 2025 Mar 15;28(1):68–75. doi: 10.1298/ptr.E10329 (PMC12047041; doi:10.1298/ptr.E10329)
Supplement: Appendix.2 — Characteristics of included studies. [file ptr-28-68-s02.pdf]

Appendix.2 Characteristics of included studies

First author	Year of publication	Country	Publication Type	Sample size	Age (mean)	Sex Man, (%)	Severity count, (%)	Injury level
Okuda A	2021	Japan	Observational study	42	NR	34 (81.0)	Complete = 35 Incomplete = 7	C4 = 15, C5 =14, C6 = 8, C7 = 5
Hachiya S	2021	Japan	Case report	1	82	1(100)	Complete = 1	C3 = 1
Murakami T	2020	Japan	Case report	1	79	0(0)	Incomplete = 1	C3–5 = 1
Miura N	2015	Japan	Case report	1	57	1(100)	Complete = 1	NR
Inoue S	2011	Japan	Case report	1	69	0(0)	Incomplete = 1	NR
Onuki T	2008	Japan	Case report	1	59	1(100)	NR = 1	C3–4 = 1
Saruyama M	2007	Japan	Case report	1	57	1(100)	Complete = 1	C6 = 1
Kiuchi S	2005	Japan	Case report	1	61	1(100)	Incomplete = 1	NR
Yamada Y	1999	Japan	Case report	1	59	1(100)	NR	C5 = 1
Lee J	1997	Japan	Case report	2	(35.0)	2 (100)	Complete = 1 Incomplete = 1	C6 = 2
Nagayama M	1996	Japan	Observational study	43	43	40 (93)	Complete = 43	C4–C8 = 43
Meredith A	2021	USA	Case report	4	(47.8)	4 (100)	NR	C3–4 = 2, C5–6 =1, C6–7 =1
Ull C	2021	Germany	Observational study	60	(27.7)	59 (98.3)	Complete = 33 Incomplete = 27	C1 = 3, C2 = 1, C3 = 2, C4 = 16, C5 = 23, C6 = 12, C7 = 3
Lee S-Y	2021	Malaysia	Case report	1	20	1(100)	NR	C3–4 = 1
Benton JA	2020	USA	Case report	1	28	1(100)	Complete = 1	C4 = 1
Marion TE	2017	Canada	Observational study	444	(47.5)	352 (79.3)	Complete = 167 Incomplete = 257	C1–4 = 139, C5–T1 = 135, T2–S5 = 154
Ijmkers S	2017	Nederland	Case report	1	62	1(100)	NR	C1–2 = 1

Riley K	2015	USA	Case report	1	34	1(100)	NR	C5–6 = 1
Oh YM	2015	Korea	Case report	1	63	1(100)	Complete = 1	T4 = 1
Wu Y-S	2014	China	Case report	1	42	1(100)	NR	C4–5 = 1
Efeoglu M	2014	Turkey	Case report	1	59	1(100)	NR	C2–7 = 1
Bartholdy K	2014	Denmark	Observational study	30	NR	22 (73.3)	NR	Cervical SCI = 24, Thoracic SCI = 6
Jain A	2013	India	Case report	1	25	1(100)	Complete = 1	T9 = 1
Dididze M	2013	USA	Observational study	35	(36.1)	27(77.1)	Complete = 35	C4 = 6, C5 = 15, C6 = 10, C7 = 4
Babu R	2013	USA	Observational study	1184	NR	NR	NR	NR
Angthong C	2011	Thailand	Case report	1	50	1(100)	Incomplete = 1	C6 = 1
Ruiz-Arango AF	2006	USA	Observational study	75	NR	NR	Complete = 25 Incomplete = 50	Above C5 = 24, Below C5 = 22, Thoracic = 19, Lumbar = 10
Chen D	1999	USA	Observational study	1649	(36.5)	NR (79)	Complete tetraplegia = NR (29.3) Complete paraplegia =NR (20.1) Incomplete tetraplegia = NR (30.7) Incomplete paraplegia = NR (18.7)	NR
Van Den Bout AH	1986	South Africa	Case report	1	22	1(100)	Complete = 1	C4 = 1
Brooke MM	1978	USA	Case report	3	33	2 (66.7)	Incomplete = 1 NR = 2	Th11 = 2, L2 =1
Mathias CJ	1976	UK	Case report	2	34	2 (100)	Complete = 4	C3/4 = 1, C4 = 1

Welply NC	1975	UK	Observational study	7	NR	7 (100)	Complete = 7	C3/4 = 1, C4 = 2, C5 = 1, NR = 3
Snow JC	1973	USA	Case report	1	20	1 (100)	NR	L4 = 1
Baker BB	1972	USA	Case report	1	44	1 (100)	NR	L1–4 = 1
Dollfus P	1965	UK	Case report & Intervention study	8	Case report: 23<br>Intervention study: NR	Case report: 1<br>Intervention study: NR	Case report: Incomplete 1<br>Intervention study: Complete = 5 Incomplete = 2	Case report : C4 = 1 Intervention study Cervical SCI = 6, Thoracic SCI = 1
Wu Y	2020	China	Case report	1	48	1(100)	NR	C1–4 = 1
Kim SW	2017	Korea	Case report	1	26	1(100)	NR	C4–7 = 1
Malmqvist L	2015	Denmark	Observational study	50	NR	37 (74.0)	Complete = 21 Incomplete = 29	C1–8 = 39, T1–5 = 5, T6–T12 = 6
Kovindha A	2014	Thailand	Case report	1	79	1 (100)	Incomplete = 1	Th11 = 1
Kumagai N	2013	Japan	Case report	1	71	1 (100)	Incomplete = 1	C4–7 = 1
Veeravagu A	2013	USA	Observational study	247019	NR	NR	NR	NR
Peyrol M	2012	France	Case report	1	46	1(100)	Complete = 1	Th2 = 1
Moerman JR	2011	USA	Observational study	106	NR	NR	NR	NR
Singh A	2010	USA	Case report	1	45	1 (100)	Incomplete = 1	C5–6 = 1
Sobiech S	2010	Poland	Case report	1	44	1 (100)	Complete = 1	C3/4 = 1
Velnar T	2010	Slovenia	Case report	1	68	1 (100)	NR	C3 = 1
Sanghvi AV	2009	India	Case report	1	47	1 (100)	Complete = 1	C7–Th1 = 1
Weant KA	2007	USA	Case report	1	25	1 (100)	NR	T4 = 1
Franga DL	2006	USA	Observational study	30	NR	NR	NR	NR
Bhuiyan MS	1998	UK	Case report	1	82	1 (100)	Incomplete = 1	T4 = 1

Tobey RE	1970	USA	Intervention study	4	NR	NR	NR	T8–L3 = 4
Gardner BP	1986	UK	Observational study	40	(40.9)	34 (85.0)	NR	C2 = 1, C3 = 1, C4 = 18, C5 = 10, C6 = 5, C7 = 1, T3 = 1, T6 = 1, T8 = 1, L1 = 1
Yamanaka T	2022	Japan	Case report	1	78	1 (100)	NR	C4–7 = 1
Chikaishi N	2022	Japan	Case report	3	(51.7)	2 (66.7)	NR	L2 = 1, C4/5=1, T5/6 =1
Funayama T	2022	Japan	Case report	1	49	1 (100)	Incomplete = 1	T1 =1
Mahanta DS	2024	India	Case report	1	25	1 (100)	Complete = 1	C5–6 = 1

NR: Not reported
